# Clinical and Radiological Outcome of Dual Plating for Proximal Humerus Fractures

**DOI:** 10.7759/cureus.33570

**Published:** 2023-01-09

**Authors:** Harsh Raithatha, Vishal S Patil, Mukund Pai, Shail Shah

**Affiliations:** 1 Orthopaedics and Traumatology, Dr. D. Y. Patil Medical College, Hospital and Research Centre, Pimpri, IND; 2 Orthopaedic Surgery, Dr. D. Y. Patil Medical College, Hospital and Research Centre, Pimpri, IND; 3 Orthopaedics, Dr. D. Y. Patil Medical College, Hospital and Research Centre, Pune, IND

**Keywords:** paavoleinen score, dash score, valgus angulation, dual plating, proximal humerus fracture

## Abstract

Introduction

Proximal humerus fractures account for approximately 4%-5% of all fractures. It accounts for approximately 45% of all humeral fractures. Proximal humerus fractures which are mostly stable or minimally displaced fractures are usually managed non-operatively with good outcomes. Displaced or unstable fractures may require reduction and stabilization. For proximal humerus fractures, conservative treatments often result in stiffness and malunion of the shoulder. In comminuted proximal humerus fractures the use of a proximal humeral internal locking system (PHILOS) only does not provide the required stable fixation which usually leads to complications such as varus collapse, malunion, anterior-posterior angulation, screw cutout, metal failure and nonunion and thus open reduction and internal fixation with dual plating are recommended for proximal humerus fractures.

Material and methods

The Institutional Ethics Committee of Dr. D. Y. Patil Vidyapeeth in Pune approved this prospective study. We included a sample size of 52 patients and conducted a study on these patients who were admitted under the Orthopedics department at Dr. D. Y. Patil Medical College and Hospital, Pune.

Results

In this study, 52 patients were treated with dual plating for proximal humerus fracture, an additional plate is used along with PHILOS. In our study, the majority of the study population belonged to > 50 years (34.6%), followed by 41-50 years (26.9%), 31-40 years (23.1%), and 21-30 years (15.4%). The mean age of the patient was 53.7 years including 33 male and 19 female patients. The majority of the patient in the study included was with RTA 40 patients and 12 patients with a history of falls from height. The fracture was classified using Neers classification, Neer type 2 fracture (23.1%), Neer type 3 fracture (46.2%), and Neer type 4 fracture (30.7%). In the current study, the mean DASH score at Baseline was 58.88±6.29, at three months was 36.23±5.05 and at six months was 31.85±4.16. The mean DASH score decreased significantly from baseline to three months to 6 months. As per the Paavolainen method, it was good among 40 (76.9%) and fair among 10 (19.2%), and poor among two (3.8%) cases. Out of 52 patients, we found varus collapse in immediate postop x-ray in two patient and screw protrusion in the glenohumeral joint in one patient.

Conclusion

Satisfactory clinical and radiological outcomes were noted. This dual mechanism prevents varus displacement of the proximal fragment, and as a result, it provides a good functional outcome with dual plates in proximal humerus fractures.

## Introduction

Proximal humerus fractures account for approximately 4%-5% of all fractures and 45% of all humeral fractures [1,2]. Thus, operative procedures for proximal humerus fractures are performed routinely in trauma care centers. Proximal humerus fractures which are mostly stable or minimally displaced are usually managed non-operatively with a good outcome. However, displaced or unstable fractures may require reduction and stabilization. For proximal humerus fractures, conservative treatments often result in stiffness and malunion [3-6]. Various modalities for surgical treatment of proximal humeral fractures include percutaneous fixation with k wires, open reduction, and internal fixation (ORIF) with plating and arthroplasty [7]. So, proximal humeral internal locking system (PHILOS) plating is being used in most of the cases in recent times. In addition, PHILOS is clinically more extensively used due to its higher elasticity, smaller size, low rigidity, and biomechanical properties like rotational stability and fixed initial angle [8]. In comminuted proximal humerus fractures, the use of only the PHILOS does not provide a required stable fixation, which usually leads to complications such as varus collapse, malunion, anterior-posterior angulation, screw cut-out, implant failure and non-union, and thus, open reduction and internal fixation with dual plating are recommended for proximal humerus fractures [9-11].

The dual plate fixation technique provides stable fixation in comminuted proximal humerus fractures. Dual plating has been used for proximal humerus fractures with metaphyseal comminution as it provides excellent anatomical reduction and neck shaft angle. Therefore, a more robust and enhanced fixation method is the dual plating technique using the locking plates (PHILOS and locking compression plate) [12].

## Materials and methods

The current research is a prospective study approved by the Institutional Ethics Committee with approval number I.E.S.C/195/2021. We included a sample size of 52 patients diagnosed with proximal humerus fractures who were admitted to the Orthopedics department at Dr. D. Y. Patil medical college and hospital, Pune. All 52 patients provided written informed consent. Following pre-anesthesia check-ups, patients who were all medically fit for surgery were scheduled for open reduction and internal fixation with dual plating for proximal humerus fractures.

Inclusion criteria

Patients who are diagnosed with proximal humerus fractures with two-part, three-part and four-part proximal humerus fractures under the age group of 18 years to 70 years were included in the study.

Exclusion criteria

Patients with open wounds, neurovascular deficits, pathological fractures, and patients with trauma for more than 21 days were excluded from the study. The patient comes to OPD with a history of shoulder pain following trauma. The patient will be assessed for any fracture with appropriate investigations. After being diagnosed with a proximal humerus fracture, a CT scan is performed to assess the fragments of the fracture. If the patient is eligible as per the inclusion criteria, he/she will be included in the study. Patients’ consent was taken and included in the study for fracture fixation with plating. The deltopectoral approach will be used for the fixation with plating and the patient will be assessed using the DASH score for clinical outcomes and radiological outcomes Paavolainen method will be used.

Study procedure

Patients on arrival at the casualty were thoroughly assessed and stabilized with a universal shoulder immobilizer. Biplanar x-rays, which include true antero-posterior of the shoulder were obtained. Special attention was given to assess function of the axillary nerve, by checking the sensation over the lateral aspect of the arm. CT Scan of the affected side was also performed to plan further management. After all pre-anesthetic fitness of patients is considered for surgery. In this study, 52 patients were assessed with the DASH scoring system post-operatively for clinical outcomes and was also assessed with the Paavoleinen score for the radiological outcomes which assesses neck shaft angle post-operatively.

Operative procedure 

**Figure 1:**
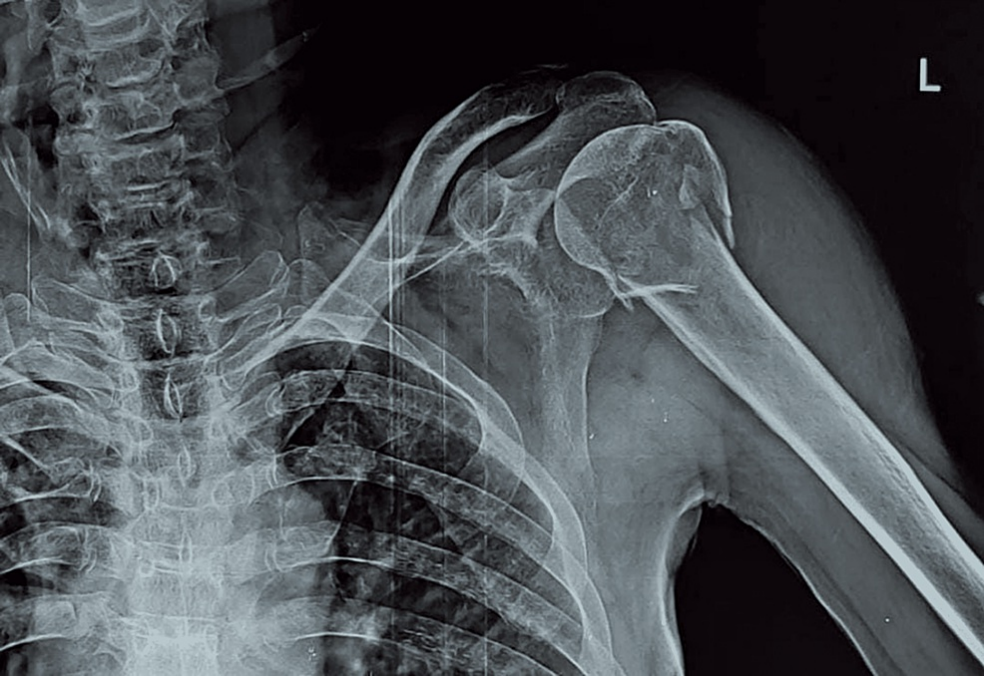
Plain radiograph antero-posterior view showing three-part proximal humerus fracture

The patient is placed in a beach chair position under general anesthesia. A deltopectoral exposure is used to expose the proximal humerus. After exposure, the fracture fragments were reduced with the best possible anatomical reduction and were maintained by Kirschner wires. The reduction was carried out by flexion and abduction of the arm. If the reduction is difficult, a k-wire is inserted as a joystick in the humeral head to rotate the head into a reduced position or placing sutures under the rotator cuff tendon (supraspinatus) also helps mobilization and reduction. The PHILOS is placed lateral to the bicipital groove 5mm distal from the tip of the greater tuberosity. The plate was first fixed with k-wires through the holes. Then valgus reduction is achieved and a static screw or osteotome was used to maintain valgus reduction. Locking cancellous screws were used to fix proximal fragments and cortical screws were used for the shaft. The rotator cuff, capsule, and subscapularis muscle tears/avulsions were repaired meticulously. An additional 3.5mm locking compression plate is placed in the anterior, medial, or posterior depending on the configuration of fracture fragments. This helps in maintaining the valgus reduction and prevents varus collapse post-operatively. Final fixation is performed with dual plating with PHILOS with other selected implants either medial, posterior, or anterior plating as per the fracture fragments. Then the postop result was assessed using the DASH score and radiological assessment is done as per the alignment and union. Figure 1 depicts AP x-ray of left-sided shoulder with three-part communited proximal humerus fracture.

**Figure 2:**
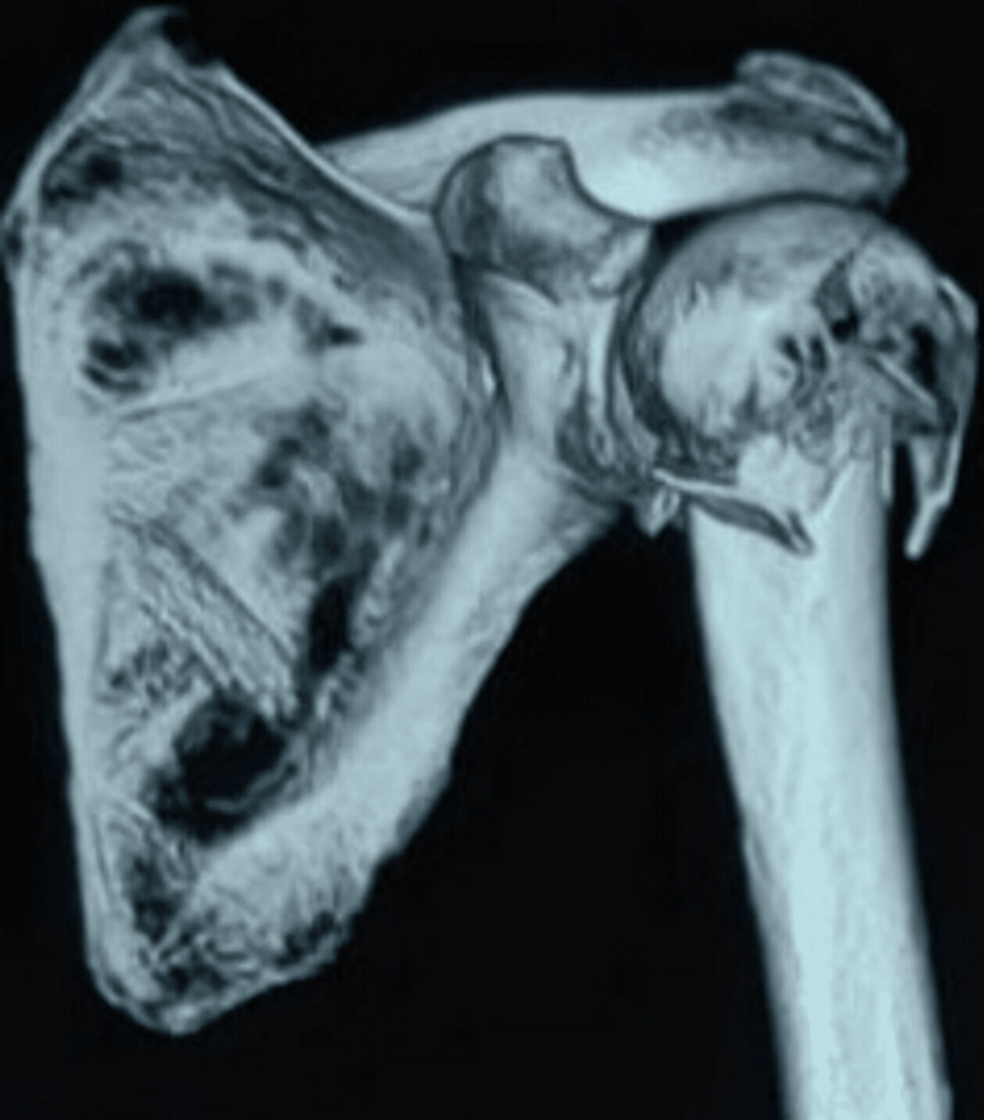
CT scan showing three-part communited proximal humerus fracture

Figure 2 shows the 3D reconstruction of three-part proximal humerus fracture.

**Figure 3:**
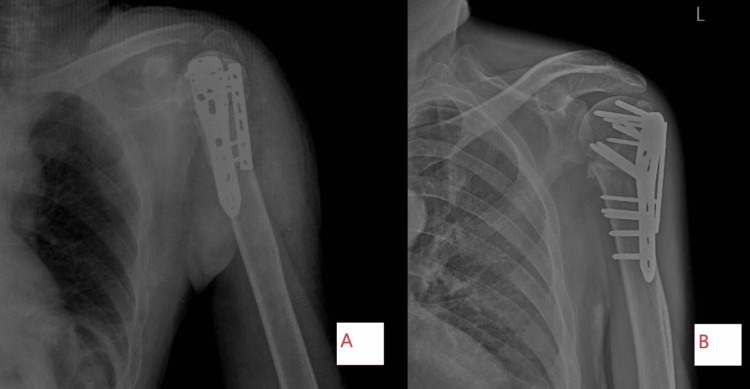
Post-operative plain radiograph antero-posterior and lateral view of dual plating for proximal humerus fracture. (A) Plain radiograph lateral view of left shoulder. (B) Plain radiograph antero-posterior view of left shoulder.

Figure 3 shows post-operative x-ray of three-part communited proximal humerus fracture operated with dual plating.

**Figure 4:**
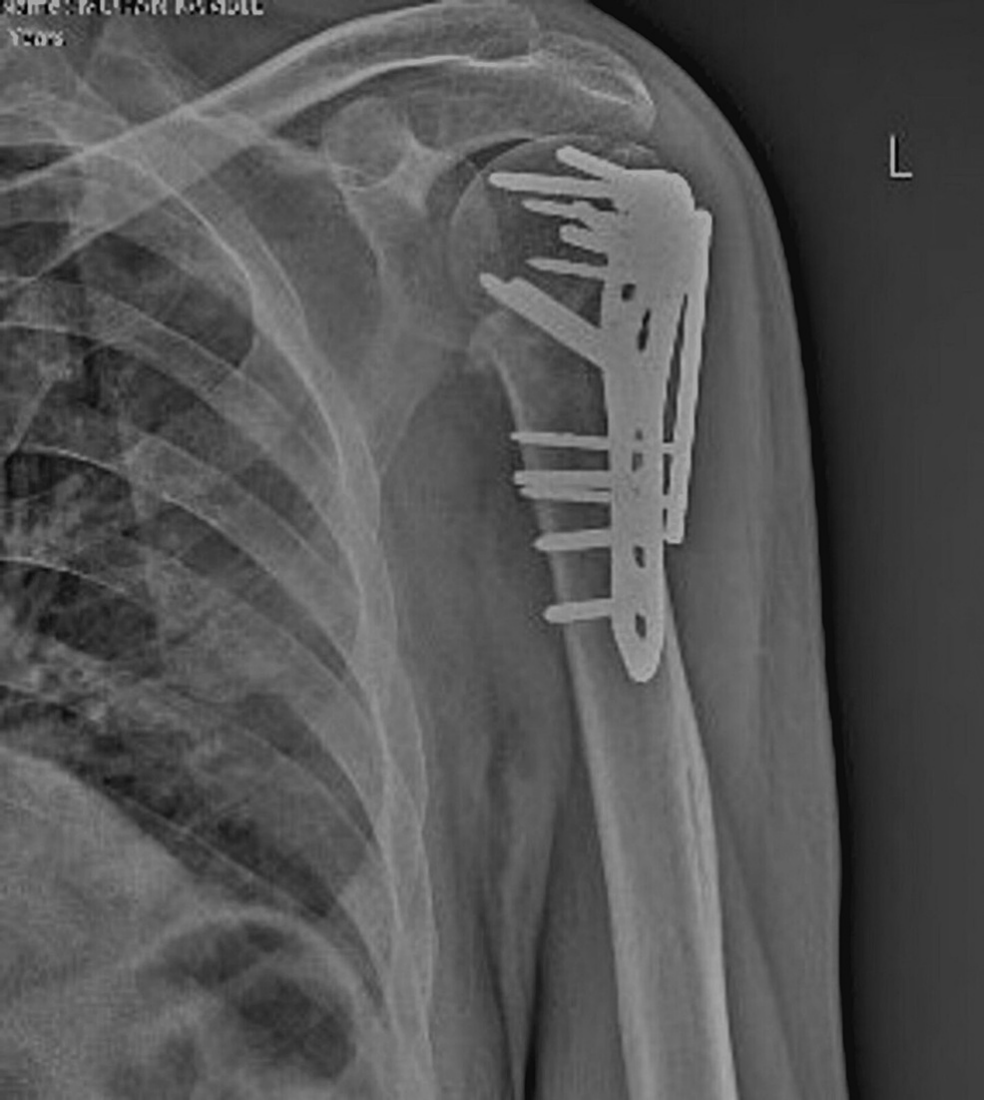
Follow-up plain radiograph antero-posterior view at three-month post-operatively

Figure 4 shows follow-up x-ray at three months post-operatively.

Post-operative rehabilitation

In all 52 patients, similar post-operative rehabilitation was performed. For the initial two days, the operated shoulder was immobilized with a Universal shoulder immobilizer. Then, the initial pendulum exercises were started for one week followed by flexion-extension exercises. For the first six weeks after surgery, only limited aided abduction was allowed up to 90 degrees [12]. Full active ROM with active exercises was started at six weeks. Radiological Assessment was performed using the Paavolainen method.

Radiographically, the fracture position was considered as good if fracture alignment and correct inclination of the humeral neck (130 degree +/- 10 degrees) was combined with good positioning of the implant, as fair if the inclination of the humeral neck ranging 100-120"; and as poor if the inclination was <100".

## Results

In this study, 52 patients were treated with dual plating for proximal humerus fracture an additional plate is used along with PHILOS to achieve valgus reduction, and an additional plate is placed anterior, medial, or posterior depending on the configuration of fracture fragments. In our study, the majority of the study population is of age > 50 years (34.6%), followed by 41-50 years (26.9%), 31-40 years (23.1%), and 21-30 years (15.4%). The mean age of the patients was 53.7 years including 33 males and 19 females. The majority of the patients in the study included were with RTA 40 patients and 12 patients with a history of falls from a certain height. The fractures were classified using the Neers classification. The findings indicated Neer type 2 fractures (23.1%), Neer type 3 fractures (46.2%), and Neer type 4 fractures (30.7%). In the current study, the mean DASH score at Baseline was 58.88±6.29, at three months was 36.23±5.05 and at six months was 31.85±4.16. The mean DASH score decreased significantly from the Baseline to three months and further at six months. As per the Paavolainen method, the score was good among 40 (76.9%), fair among 10 (19.2%), and poor in two (3.8%) cases. Out of 52 patients, we found varus collapse in immediate postop x-ray in two patients and screw protrusion in the glenohumeral joint in one patient. Delto-pectoral approach was used for fixation for all patients. The patient was followed up until the union of fracture or until any complication arose and planned further management was. The average follow-up ranged from zero to six months post-operatively. Patients were restricted for mobilization following fracture union and until the patient returned to their normal physical activities. The patient was called for a follow-up on an OPD basis and the average union time was 12-16 weeks. Over radiological assessment postoperative was found to be good and fair, only in two patients, while the Paavoleinin score was found to be poor. The average neck shaft angle was 128 degrees.

## Discussion

Proximal humerus fractures have always been challenging, especially in comminuted and osteoporotic fractures. Such a fracture usually requires operative management to achieve anatomical reduction and restore function. Osteoporotic patients are usually with low energy trauma and may exhibit complex fracture patterns. Thus, it is very difficult to achieve proper anatomical reduction and the screw purchase is also very high and which accounts for high failure rates [12]. Various modalities of treatment are being attempted for proximal humerus fractures such as percutaneous screw fixation, k-wiring, Intramedullary nailing, and hemiarthroplasty of the shoulder joint. But on long follow-up complications such as malunion, stiffness, nonunion, and avascular necrosis of the humeral head are also noted at very higher rates as compared to open reduction and internal fixation by plating provides relatively good anatomical reduction and help in early mobility [13]. In the study by Laux [14], it was stated that proximal humerus fixation remains challenging for many surgeons. To restore the anatomical reduction and to prevent varus collapse additional support is required. In our study, we used a dual plate with valgus angulation fixation which provided excellent results in overall clinical and radiological assessments. In a study by Tristan [15], the locking plates significantly improved the fixation of proximal humerus fractures, and also identified fractures with marked medial comminution and varus deformity. Loss of reduction and fixation failure were the most commonly reported complications. So, the additional support by additional locking plates helped to prevent varus collapse and fixation failure which helps to start mobilization early with a significant reduction in pain and improved arc of motion as compared to fixation done with other various modalities of fixation. Choi et al. [16] state that PHILOS alone cannot be effective in stable fixation for proximal humerus fracture. An additional plate is required to maintain adequate reduction and varus collapse prevented by dual plating. The overall findings for fixation of proximal humerus fracture with locking plates provided fairly good anatomical reductions and significantly reduced complications like screw cutout, avascular necrosis of the humeral head, a reduced arc of motion, persistent pain over a longer period, and fixation failure. In additional usage of an additional locking plate according to fracture fragment in comminuted fractures help to prevent anteroposterior collapse, varus collapse, and fixation failure and allowed to achieve the valgus angulation reduction.

## Conclusions

The management of proximal humerus fractures remains a challenging task for surgeons owing to the diversity of fracture patterns and the varying bone quality, particularly in the geriatric population with osteoporosis. The dual mechanisms mentioned provide stable fixation with valgus angulation, which further reduces the incidence of screw pullout, and failure of fixation and also prevents varus collapse. Thereby providing a good functional and radiological outcome with dual plating in proximal humerus fractures.
